# An l-isosorbide-based reactive chiral dopant with high helical twisting power for cholesteric liquid crystal polymers reflecting left-handed circularly polarized light†

**DOI:** 10.1039/d4qo01672f

**Published:** 2024-10-09

**Authors:** Ramazan Umut Dinc, Johan Lub, Augustinus J. J. Kragt, Albert P. H. J. Schenning

**Affiliations:** a Laboratory of Stimuli-responsive Functional Materials and Devices (SFD), Department of Chemical Engineering and Chemistry, Eindhoven University of Technology P.O. Box 513 5600 MB Eindhoven The Netherlands a.p.h.j.schenning@tue.nl

## Abstract

For visible light reflective cholesteric liquid crystal polymers, reactive chiral dopant enantiomers with high helical twisting power are attractive. However, a chiral dopant for reflecting left-handed circularly polarized light has been missing so far. Here, we report the synthesis of a reactive, left-handed, l-isosorbide-based chiral dopant with a high helical twisting power of −48 μm^−1^ that can be used in visible light reflective cholesteric liquid crystal polymers. The right handed dopant enantiomer was also synthesized, showing a helical twisting power of +63 μm^−1^.

## Abstract 2

We report an l-isosorbide-based reactive chiral dopant with high helical twisting power for cholesteric liquid crystal polymers reflecting left-handed circularly polarized light.

## Graphical abstract

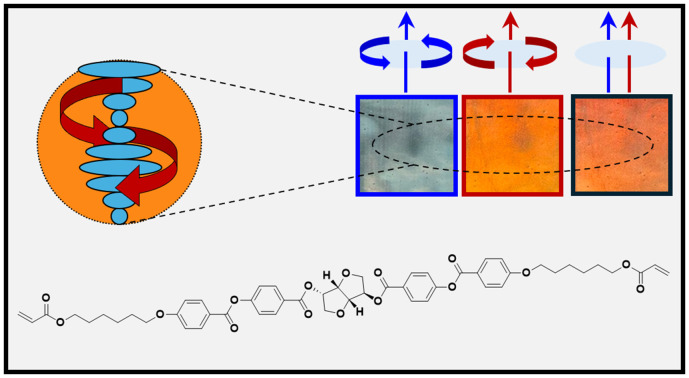

## Introduction

Cholesteric liquid crystal (CLC) polymers are intriguing photonic materials that can reflect circularly polarized light.^1^ Adding a chiral dopant molecule to a nematic liquid crystal induces helical chirality, resulting in a chiral nematic or CLC phase, which produces structural color. Polymers are often fabricated by using an acrylate-based reactive mesogenic CLC mixture that is subsequently photopolymerized. CLC polymers have a wide range of applications,^2,3^ such as sensors,^4,5^ soft structural colored actuators,^6–8^ anti-counterfeit devices,^9,10^ decoration,^11,12^ and smart windows.^13,14^

**Scheme 1:**
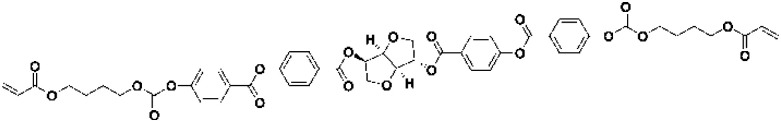
Chemical structure of the d-isosorbide-based reactive chiral dopant LC756.

The structural color of CLC polymers depends on the helical pitch, the distance of a full turn of the reactive mesogens, and the average refractive index, which is typically around 1.6.^15,16^ The pitch length is inversely proportional to the helical twisting power (HTP), enantiomer excess (ee), and the concentration of the reactive chiral dopant. A high HTP value is attractive to maintain the nematic liquid crystalline phase, since many of the chiral dopants do not show liquid crystalline properties themselves. Hence, a low concentration of the chiral dopant can be used to achieve visible light reflectance^17^ without affecting the liquid crystal behavior. Nowadays, the reactive d-isosorbide-based chiral dopant LC756 (Scheme 1) is the most commonly used due to its commercial availability and high HTP (83 μm^−1^),^18^ which allows it to be used in low concentrations for visible light reflectance.^19,20^d-Isosorbide is a biobased bicyclic compound derived from starch. Typically, around 5 wt% of the dopant is needed to obtain a CLC polymer that reflects in the visible light region. However, LC756 contains carbonate groups that are labile during the base-catalyzed thiol–acrylate Michael addition reaction, often used to prepare stimuli-responsive CLC polymers.^21^

Theoretically, the maximum reflectance of a CLC polymer can reach up to 50% of unpolarized light due to the intrinsic handedness of the chiral dopant.^22^ Hence, CLCs doped with the d-isosorbide-based chiral dopant LC756 can only reflect right-handed circularly polarized (RCP) light. In order to achieve near 100% reflectance of unpolarized light or selectively reflect left-handed circularly polarized (LCP) light, a reactive left-handed chiral dopant with a high HTP is required. Such a dopant has not been reported so far. Reactive chiral dopants with moderate HTPs have been used to fabricate left-handed CLC polymers.^17^

**Scheme 2:**
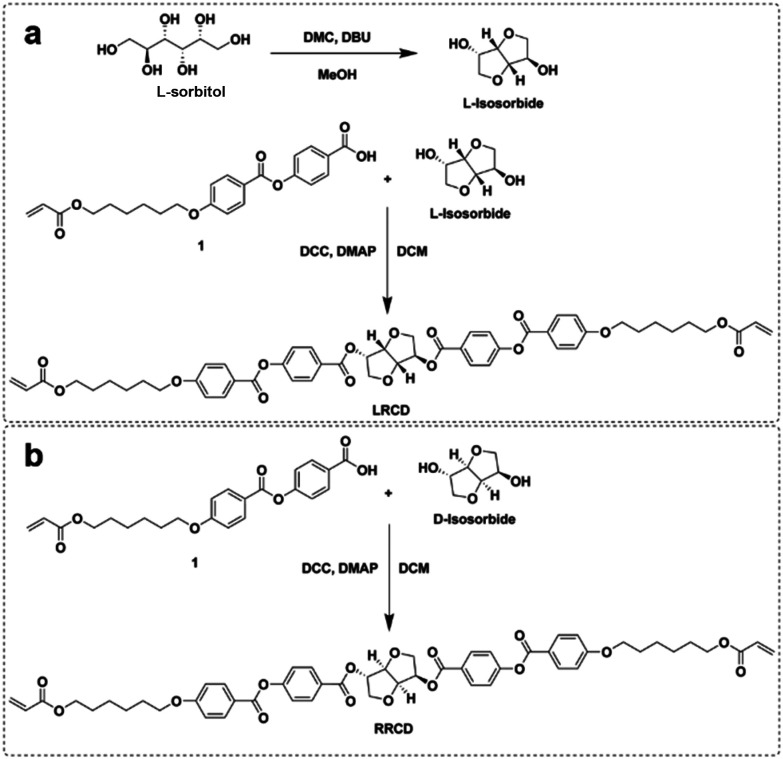
a) Synthesis of l-isosorbide and LRCD. (b) Synthesis of RRCD.

Here, we report the synthesis of a reactive left-handed reactive chiral dopant derived from l-isosorbide (LRCD, Scheme 2a). The dopant is similar to LC756 but does not contain carbonate groups and it is less laborious to synthesize.^23^ We synthesize LRCD in two steps using l-sorbitol and acrylate benzoic acid derivative 1. In this way, we circumvent laborious intermediate steps of protection/deprotection of alcohol groups.^24^ In the first step, l-sorbitol is converted into l-isosorbide and in the second step l-isosorbide is reacted with 1 to yield LRCD. The right-handed RRCD enantiomer is also synthesized in the same way using commercially available d-isosorbide and 1 (Scheme 2b ^25^).

## Experimental

### Materials

All commercial chemicals and solvents were used as received unless stated otherwise. d-Sorbitol, l-sorbitol and 3,6-dioxa-1,8-octane-dithiol (DODT) were obtained from Tokyo Chemical Industries (Japan). d-Isosorbide was obtained from Alfa Aesar (USA). Diacrylate mesogens 2-methyl-1,4-phenylene bis(4-((6-(acryloyloxy)hexyl)oxy)benzoate) (C6M) and 2-methyl-1,4-phenyl-ene bis(4-(3-(acryloyloxy)propoxy)benzoate) (C3M) are obtained from Daken Chemical (China). Deuterated chloroform (CDCl_3_), dimethyl sulfoxide-d_6_ (DMSO-d_6_), dimethyl carbonate (DMC), 1,8-diazabicyclo(5.4.0)undec-7-ene (DBU), *N*,*N*′-dicyclohexylcarbodiimide (DCC), 4-(dimethylamino)pyridine (DMAP), and α-methylbenzylamine (α-MBA) were obtained from Sigma-Aldrich (USA). BASF Lumogen S1065 was obtained from BASF (Germany). Omnirad TPO was obtained from IGM Resins (The Netherlands). Surfactant BYK-361-N was obtained from BYK Additives & Instruments (Germany). Dichloromethane (DCM), methanol (MeOH), ethanol (EtOH), isopropanol (IPA), tetrahydrofuran (THF), chloroform (CHCl_3_), and cyclopentanone were obtained from BioSolve (The Netherlands). Biaxially stretched polyethylene terephthalate (PET) Melinex® foil was obtained from DuPont (USA).

### Synthesis of l-isosorbide

2.0 mL of MeOH was put into a round bottom flask. Subsequently, 1.0 g of l-sorbitol (5.5 mmol, 1 mol eq.), 3.697 mL of DMC (44 mmol, 8 mol eq.), and 0.041 mL of DBU (0.027 mmol, 0.05 mol eq.) were refluxed under an Ar atmosphere at 300 RPM for 24 hours. A brown/yellow product^26^ was obtained after evaporation. This product was eluted over a SiO_2_ column with an 8 : 1 volume ratio of DCM : MeOH for further purification. 0.25 grams of l-isosorbide (31% yield) were obtained as a viscous oil. ^1^H-NMR (DMSO, 400 MHz, Me_4_Si, *δ* in ppm, J in Hz): *δ* 5.12 (d, *J* = 3.7 Hz, 1H), 4.72 (d, *J* = 6.7 Hz, 1H), 4.35 (t, *J* = 4.4 Hz, 1H), 4.24 (dt, *J* = 4.2, 0.9 Hz, 1H), 4.09 (dtd, *J* = 8.2, 6.7, 4.7 Hz, 1H), 4.04 (td, *J* = 3.5, 1.7 Hz, 1H), 3.78–3.65 (m, 3H), 3.25 (t, *J* = 8.2 Hz, 1H). ^13^C-NMR (DMSO, 101 MHz, Me_4_Si, *δ* in ppm): *δ* 88.21, 81.72, 76.16, 75.72, 72.72, 71.30. GC-MS: single peak, calcd for [C_6_H_10_O_4_]: 146.06 *m*/*z*, found 146.1.

### Synthesis of LRCD and RRCD

0.62 g of 1,3-dicyclohexylcarbodiimide (DCC, 3.0 mmol, 2 mol eq.) was added to a mixture of 0.20 g of l-isosorbide (1.3 mmol, 1 mol eq.), 1.1 g of 4-(4-(6-acryloyloxyhexyloxy)benzoyloxy)benzoic acid (compound 1, 0.26 mmol, 2 mol eq.), 0.0167 g of 4-(dimethylamino)pyridine (DMAP, 0.14 mmol, 0.1 mol eq.) and 10 mL of dichloromethane. The mixture was stirred in an ice bath under an Ar atmosphere. After stirring for another 24 hours at room temperature, the mixture was filtered over a thin layer of a silica pad and the solvent was evaporated.^27–29^ 2.0 grams of the product (LRCD, 38% yield) were obtained as a white solid after elution over a SiO_2_ column with a 1 : 2 volume ratio of ethyl acetate :  *n*-heptane. Melting point (mp) = 80 °C. ^1^H NMR (CDCl_3_, 400 MHz, Me_4_Si, *δ* in ppm, *J* in Hz): 8.16 (d, *J* = 8.8, 2H), 8.14 (d, *J* = 8.9, 2H), 8.13 (d, *J* = 8.9, 2H), 8.10 (d, *J* = 8.8, 2H), 7.32 (d, *J* = 8.8, 2H), 7.30 (d, *J* = 8.7, 2H), 6.97 (d, *J* = 8.9, 2H), 6.98 (d, *J* = 8.9, 2H), 6.41 (dd, *J*_1_ = 17.3, *J*_2_ = 1.5, 2H), 6.13 (dd, *J*_1_ = 17.3, *J*_2_ = 10.4, 2H), 5.82 (dd, *J*_1_ = 10.4, *J*_2_ = 1.5, 2H), 5.51 (br, 1H), 5.44 (q, *J* = 5.4, 1H), 5.08 (t, *J* = 5.1, 1H), 4.69 (d, *J* = 4.7, 1H), 4.18 (t, *J* = 6.7, 4H), 4.13 (br, 2H), 4.07 (m, 2H), 4.06 (t, *J* = 6.4, 2H), 4.05 (t, *J* = 6.5, 2H), 1.85 (p, *J* = 6.6 Hz, 2H), 1.85 (p, *J* = 6.6 Hz, 2H), 1.73 (p, *J* = 6.8 Hz, 4H), 1.60–1.40 (m, 8H). ^13^C-NMR (CDCl_3_, 101 MHz, Me_4_Si, *δ* in ppm, *: CH or CH_3_, #: CH2): 166.42, 165.34, 165.00, 164.43, 164.40, 163.80, 155.31, 155.24, 132.53*, 131.52*, 131.49*, 130.69#, 128.68*, 126.99, 126.94, 122.10*, 121.20, 121.16, 114.50*, 86.32*, 81.29*, 78.65*, 74.73*, 73.62#, 70.90#, 68.25#, 64.57#, 29.10#, 28.67#, 25.84#, 25.82#. MALDI-TOF-MS: [M + Na]^+^ calcd for C_52_H_54_O_16_; 957.33 *m*/*z*, found 957.205.

RRCD was prepared in the same way as LRCD using d-isosorbide instead of l-isosorbide with a total yield of 47%. Purification was performed by crystallization from a 20 : 80 mixture of DCM and IPA. Melting point (mp) = 80 °C. The ^1^H-NMR and ^13^C-NMR spectra yielded the same signals as LRCD (Fig. S6 and S7†). MALDI-TOF-MS: [M + Na]^+^ calcd for C_52_H_54_O_16_; 957.33 *m*/*z*, found 957.205.

### Preparation of CLC polymer coatings

920.8 mg of BASF Lumogen S1065 (92.1 wt%, as the LC monomer), 56.2 mg of LRCD (5.6 wt%), and 23.0 mg of Omnirad TPO (5.5 mol% of total mol of acrylates, as the initiator) were added to a 20 mL amber vial. For RRCD coating, the concentration of the chiral dopant was set to 4.7 wt%. To decrease the viscosity of the coating for the ease of bar-coating, 736.8 μL of cyclopentanone (70 wt% of dry monomer) was added to the same vial. Afterward, to induce planar anchoring on the air/coating interface, 52.6 μL (1 wt% solution in cyclopentanone) of surface leveling agent BYK-361-N was added to the same batch. The mixture was stirred at 80 °C and 300 RPM for 30 min before coating. The CLC mixtures were coated onto a PET substrate *via* the bar-coating method. Just before coating, the PET substrate was cleaned with IPA and dried with N_2_. The total gap for the coatings was set to 15 μm. Depending on the size of the coating, the required amount of the CLC mixture was put in front of a 4-sided applicator and the coating was done by moving the 4-sided applicator on the PET substrate at a constant speed *via* a bar coater to distribute the CLC mixture on the substrate evenly. The coatings were put in an oven at 110 °C for 5 minutes to evaporate the solvent and align the LC mesogens. Within 2 minutes of oven heat treatment, the coatings were put on a UV exposing conveyor belt and they were photopolymerized at 3500 mW cm^−2^ UV (365 nm) intensity for 5 seconds. After the free radical photopolymerization, the coatings were safe to touch without any disruption to the coating surface, which supports full polymerization.

## Results and discussion

Optical rotation measurements revealed the specific optical rotation of l-sorbitol as −1.3 ± 0.2° 100 mL g^−1^ dm^−1^, while d-sorbitol showed an optical rotation of 1.7 ± 0.2° 100 mL g^−1^ dm^−1^ (Fig. S1†). Assuming that d-sorbitol is enantiomerically pure since it is a catalytic hydrogenation product of the precursor d-glucose which can be sourced from nature,^30^ the calculated enantiomer excess percentage, ee%, of l-sorbitol is 76 ± 16%. Most likely, l-sorbitol contains d-sorbitol since it is chemically pure according to ^1^H-NMR (nuclear magnetic resonance) measurements (Fig. S2†) and GC (gas chromatography) analysis.

The synthesis of l-isosorbide was carried out according to a modified literature procedure by a dimethyl carbonate (DMC)-mediated double cyclization reaction using diazabicyclo(5.4.0)undec-7-ene (DBU) as a base.^26^l-Isosorbide was obtained in a yield of 31% after column chromatography and fully characterized. ^1^H-NMR and ^13^C-NMR showed the same chemical shifts as the commercial d-isosorbide (Fig. S3 and S4†). GC/MS (mass spectrometry) analysis showed a single peak at 4.68 min, with a mass fragmentation pattern corresponding to isosorbide (Fig. S5†). Optical rotation measurements revealed a specific optical rotation of l-isosorbide of −185 ± 0.4° 100 mL g^−1^ dm^−1^, while d-isosorbide showed an optical rotation of −260.20 ± 0.04° 100 mL g^−1^ dm^−1^ (Fig. S9†). Assuming that d-isosorbide has an ee of 100%, the ee% of l-isosorbide is approximately 71% that of d-isosorbide.

**Fig. 1:**
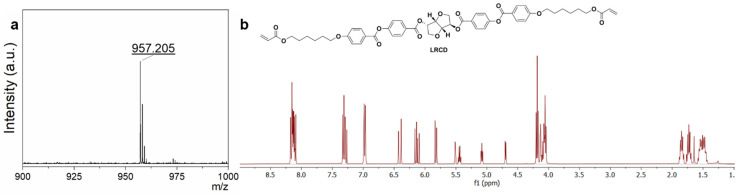
(a) MALDI-TOF-MS measurement of LRCD. (b) The ^1^H-NMR spectrum of LRCD in CDCl_3_.

After the synthesis of l-isosorbide, the l-isosorbide-based chiral dopant LRCD was obtained by a Steglich esterification reaction^27^ using l-isosorbide and 1 (Scheme 2a). Compound 1 was synthesized as reported in the literature.^27–29^ We also synthesized RRCD using 1 and commercial d-isosorbide (Scheme 2b). The ^1^H-NMR and ^13^C-NMR spectra of LRCD showed the same chemical shifts as those of RRCD, indicating an enantiomeric relationship between the two compounds (Fig. 1b and Fig. S6 and S7†). MALDI-TOF-MS (matrix-assisted laser desorption/ionization time-of-flight) analysis of LRCD (Fig. 1a) and RRCD (Fig. S8†) revealed a mass peak at 957.205 corresponding to the [M + Na]^+^ adduct. The specific optical rotations for RRCD and LRCD were found as −263.0 ± 0.5° 100 mL g^−1^ dm^−1^ and 214 ± 1° 100 mL g^−1^ dm^−1^, respectively. Assuming that RRCD has an enantiomer excess of 100%, the ee of LRCD is approximately 81% (Fig. S9†).

**Fig. 2:**
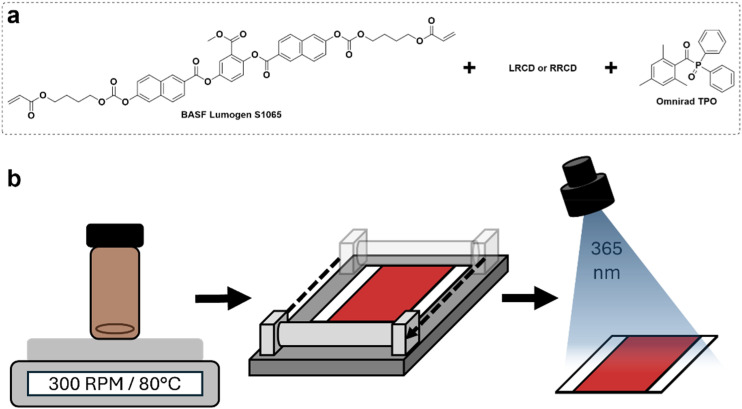
(a) The CLC mixture components. (b) The schematic of the coating procedure of the CLC mixture on a PET substrate, followed by free radical photopolymerization of diacrylates.

We prepared visible light reflecting CLC polymer coatings using reactive LRCD and RRCD as dopants on a PET substrate (Fig. 2). We selected BASF Lumogen S1065 as the nematic LC monomer due to its high birefringence (Δ*n* = 0.2096).^31^ For the LRCD polymer coating, the chiral dopant (5.6 wt%) was dissolved in cyclopentanone containing a reactive nematic mesogen (92.1 wt%) and photoinitiator (2.3 wt%) CLC mixture. The CLC mixture was bar-coated onto a PET substrate. After evaporating the solvent, the CLC mixture was photopolymerized using intense UV light, resulting in an orange reflective polymer coating. The same coating procedure was used to produce the RRCD polymer coating, only changing the chiral dopant from LRCD to RRCD and the concentration to 4.7 wt%.

**Fig. 3:**
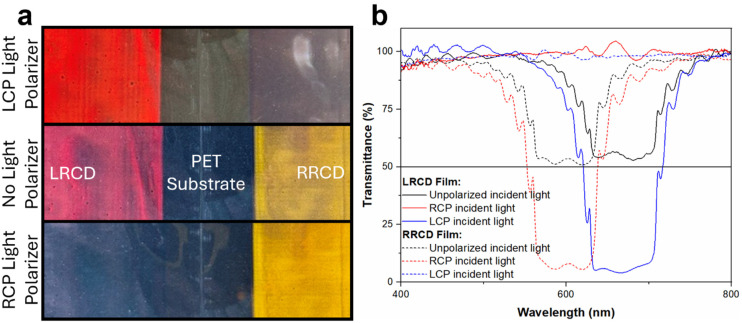
(a) Visible light reflective CLC polymer coatings on PET substrates, observed through different circularly polarized light polarizers. (b) The UV-Vis-NIR transmittance spectra of the same coatings with different circularly polarized incident light.

We analyzed the LRCD and RRCD polymer coatings by UV-Vis-NIR spectroscopy (Fig. 3). The transmission spectrum of the LRCD coating shows a reflection peak at 670 nm corresponding to an orange color. When the incident light is unpolarized, the coating transmits 50% of the incident light. When using LCP light, no light was transmitted, while in the case of incident RCP light, all light was transmitted. These results indicate that the coating has a left-handed helical structure and reflects LCP incident light. Indeed, the coating is not visible with an RCP light polarizer but the orange color is visible with an LCP light polarizer (Fig. 3). The RRCD coating has a reflection band at 603 nm and reflected only RCP light as expected. Since LRCD and RRCD are enantiomers, their HTP values must be identical in magnitude but antithetical in sign. Theoretically, when the ee% values of the chiral dopant enantiomers are the same, CLC mixtures with the same concentrations of chiral dopants should have reflection peaks at the same wavelength. Assuming that the average refractive index is 1.6 for the reactive nematic liquid host, the pitch lengths were calculated to be 419 nm and 377 nm for the LRCD coating and RRCD coating, respectively. The apparent HTPs of LRCD and RRCD were calculated to be −48 and +63 μm^−1^, respectively. The ratio of these apparent HTPs of LRCD to RRCD is 76%, which is in agreement with the calculated ee% of LRCD, based on optical rotation measurements.

## Conclusions

We reported the two-step synthesis of a left-handed l-isosorbide reactive chiral dopant, LRCD, with a high HTP core. The chemical structure was confirmed by ^1^H-NMR and ^13^C-NMR spectra, GC/MS, and MALDI-TOF-MS analyses. Its enantiomer, RRCD, was also synthesized in the same manner and fully characterized. The LRCD CLC polymer coating selectively reflected LCP light. The HTP of LRCD is −48 μm^−1^, which is in the same range as the RRCD enantiomer, +63 μm^−1^. This novel left-handed reactive chiral dopant could be used for many applications, including energy saving, anti-counterfeit, decoration, and other applications that need both RCP and LCP reflections. This reactive chiral dopant can be used not only in CLC polymer coatings having static optical properties but also in thermochromic light reflective coatings (Fig. S10†). Depending on the desired properties, our synthetic procedure can be easily altered to prepare other reactive isosorbide-based dopant enantiomers with a high helical twisting power.

## Author contributions

R. Umut Dinc: writing – original draft; conceptualization (equal); investigation; visualization; and writing – review & editing (equal). Augustinus J. J. Kragt: supervision (supporting); conceptualization (equal); and writing – review & editing (supporting). Albert P.H.J. Schenning: funding acquisition (lead); resources (lead); conceptualization (equal); supervision (equal); and writing – review & editing (supporting). Johan Lub: conceptualization (equal) and writing – review & editing (supporting).

## Data availability

The ESI including characterization, equipment, and additional analyses and graphs is available free of charge on the RSC Publications website. The database of this publication is available free of charge at https://zenodo.org/doi/10.5281/zenodo.13740657.†

## Conflicts of interest

There are no conflicts to declare.

## Supplementary Material

QO-011-D4QO01672F-s001
