# Supplementary material for: An l-isosorbide-based reactive chiral dopant with high helical twisting power for cholesteric liquid crystal polymers reflecting left-handed circularly polarized light
Source: Org Chem Front. 2024 Oct 9;11(24):7053–8. doi: 10.1039/d4qo01672f (PMC11492181; doi:10.1039/d4qo01672f)
Supplement: QO-011-D4QO01672F-s001 [file QO-011-D4QO01672F-s001.pdf]

This journal is © the Partner Organisations 2024

Supporting Information: Additional experimental details, methods and graphs

Characterization & Equipment

^{1}H-Nuclear magnetic resonance (NMR) samples were prepared by taking 2 mg of the dry compound and dissolving it in either 0.6 mL $CDCl_3$ or DMSO-d_6. For ^{13}C-NMR, the dissolved amount increased to 20 mg of the dry compound. The spectra were measured using a Bruker Avance Core III 400MHz spectrometer and the signals were analyzed using MestRenova software. The melting point (mp) were determined using Discovery DSC2500 by TA Instruments. Gas chromatography/mass spectrometry (GCMS) samples were prepared by dissolving 1 mg of dry compound in 1 mL $CHCl_3$. The spectra were measured using Shimadzu GCMS-QP2020 and the signals were analyzed using post-run analysis software. Matrix-assisted laser desorption/ionization - time of flight (MALDI-TOF) samples were prepared by dissolving 1 mg of the dry compound in 1 mL THF. The spectra were measured using Bruker Daltonics Autoflex and the signals were analyzed using Bruker Daltonics flexAnalysis software. Optical rotation measurement samples were prepared by dissolving compounds in water, EtOH, or $CHCl_3$ depending on solubility. The optical rotations of the compounds were measured using a Jasco P-2000 polarimeter. The optical rotation data was gathered using Jasco Spectra Manager™ software and the standard deviation of the measurement reported as is. The specific optical rotations were calculated in Origin by OriginLab.

Preparation of thermochromic light reflective CLC coating:

The synthesis of the thermochromic light reflective CLC coatings were done according to literature[1]. For a 0.5 g of mixture, 329.4 mg C6M and 96.1 mg C3M and 15 mg of **LRCD** were added to a solution of 2.5 mL DCM containing 55.2 µL DODT in a 25 mL brown vial. Subsequently, 2.58 µL α-MBA was added to mixture to initiate Michael-thiol addition reaction and the mixture was stirred at 35°C for overnight. Then the cap of the vial opened and the solvent was evaporated at 55°C overnight. The mesogen molar ratio of C6M: C3M was set to 3. The molar ratio of mesogen:DODT was set to 2. To decrease the viscosity of coating for the ease of bar-coating, 263.2 µL cyclopentanone (50 wt.% of dry oligomer mixture) was added into the same vial. Afterward, to induce planar anchoring on the air/coating interface, 26.3 µL (1 wt.% solution in cyclopentanone) surface leveling agent BYK-361-N was added to the same batch. The mixture was stirred at 80°C, 300 RPM for 30 min just before coating. The CLC mixtures were coated onto PET substrate via the bar-coating method. Just before coating, the PET substrate was cleaned with IPA and dried with N_2. The total gap for the coating was set to 15 µm. The coating was put in an oven at 60°C for 30 minutes to evaporate the solvent and align the LC mesogens.

Specific optical rotation measurements of D- and L-Sorbitol

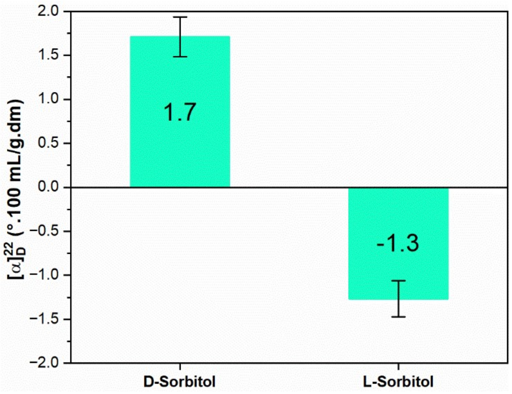

Figure S1: Specific optical rotation measurements of D- and L-Sorbitol. (C=0.100 for D-Sorbitol, C=0.092 for L-Sorbitol; both in water)

^{1}H-NMR spectra of L-Sorbitol and D-Sorbitol

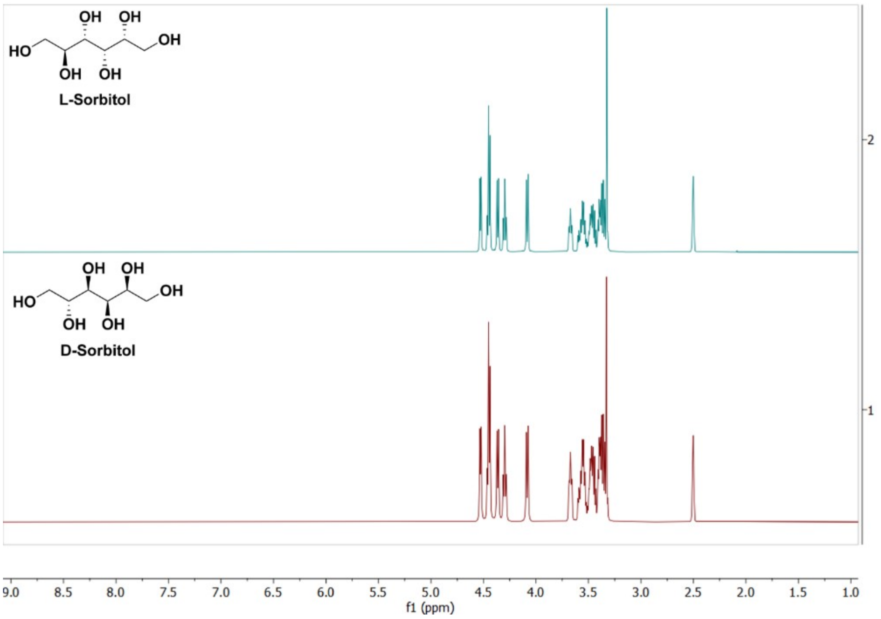

Figure S2: ^{1}H-NMR spectra of L-Sorbitol and D-Sorbitol in DMSO-d_6.

^{1}H-NMR spectra of L-Isosorbide and commercial D-Isosorbide

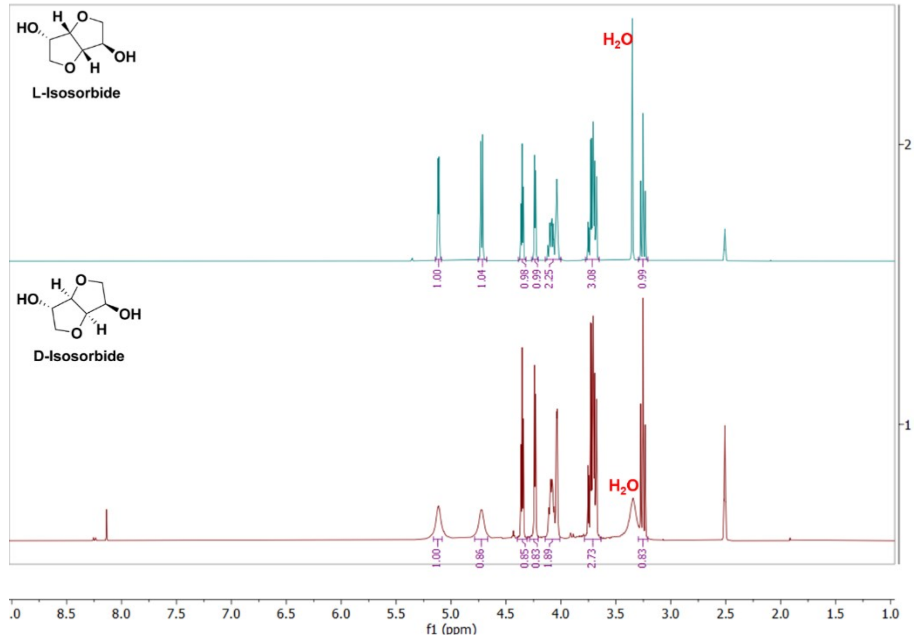

Figure S3: ^{1}H-NMR spectra of L-Isosorbide and commercial D-Isosorbide in DMSO-d_6.

^{13}C-NMR spectra of L-Isosorbide and commercial D-Isosorbide

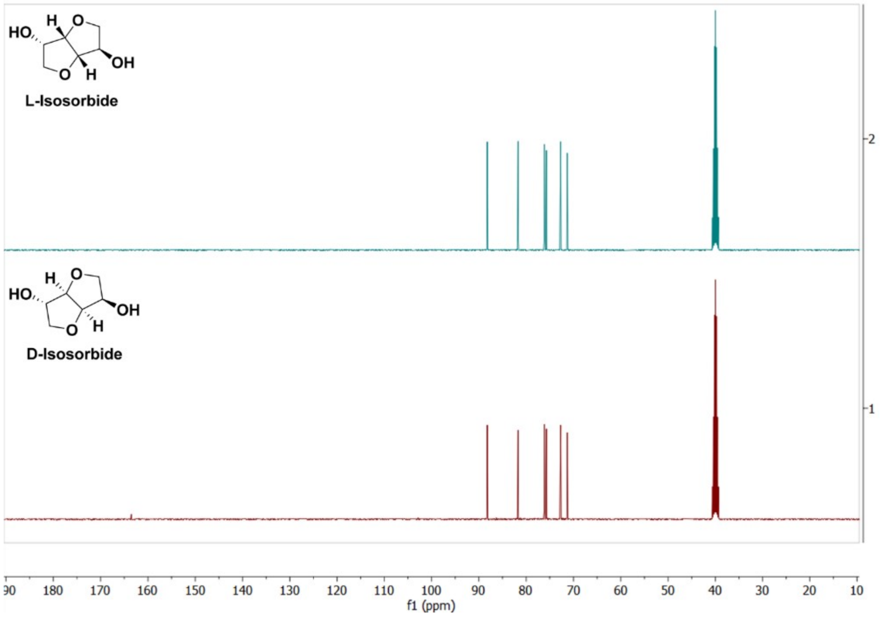

Figure S4: ^{13}C-NMR spectra of L-Isosorbide and commercial D-Isosorbide in DMSO-d_6.

GC/MS analyses of L-Isosorbide and commercial D-Isosorbide

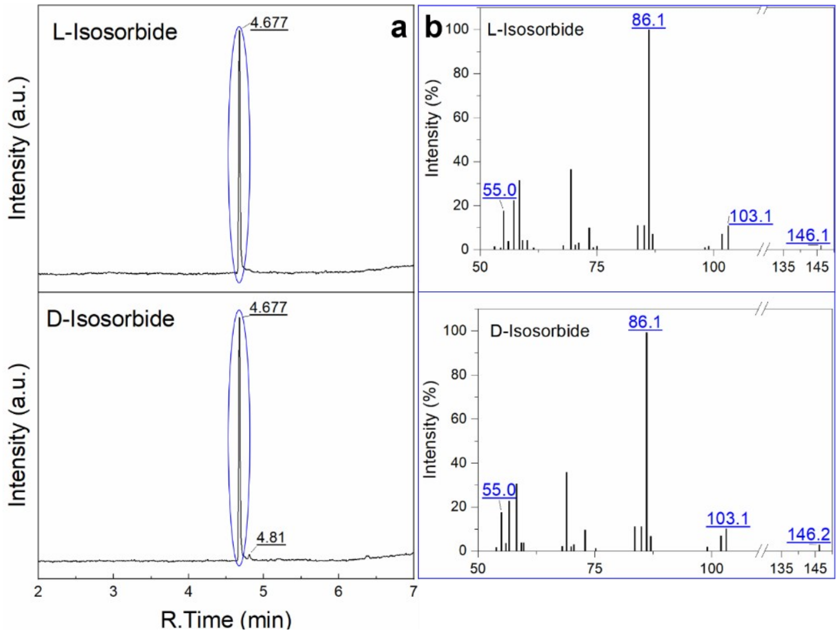

Figure S5: a) GC spectra of L-Isosorbide and D-Isosorbide, both showing an intense peak at 4.68 min. b) The mass spectra of L-Isosorbide and D-Isosorbide at 4.68 min.

^{1}H-NMR spectra of LRCD and RRCD

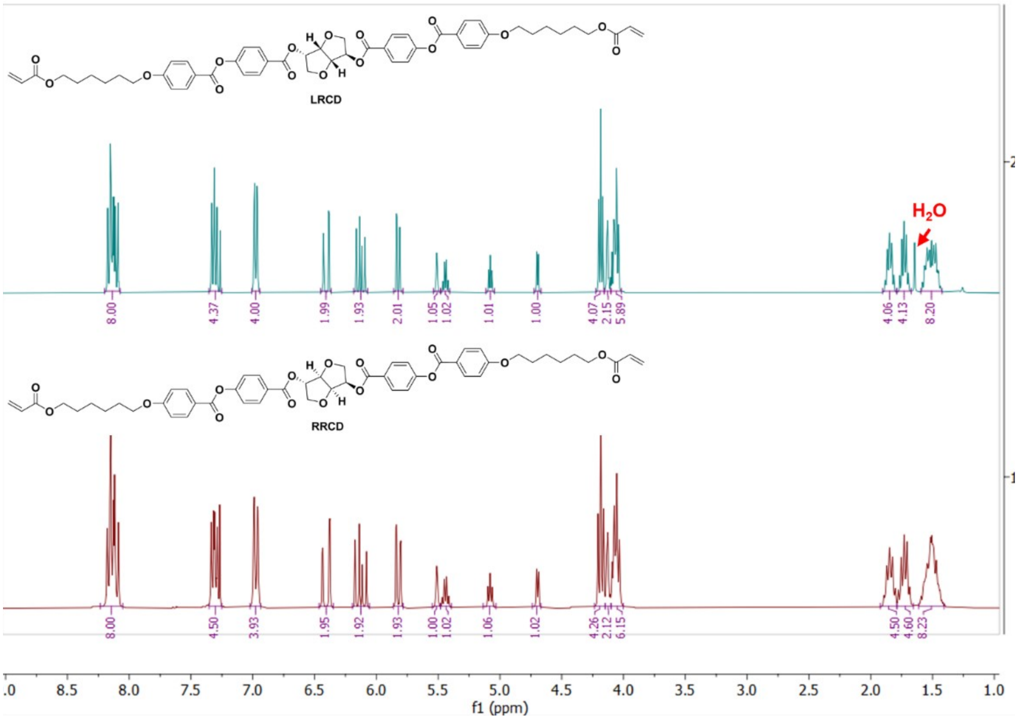

Figure S6: ^{1}H-NMR spectra of LRCD and RRCD in $CDCl_3$.

^{13}C-NMR spectra of LRCD and RRCD

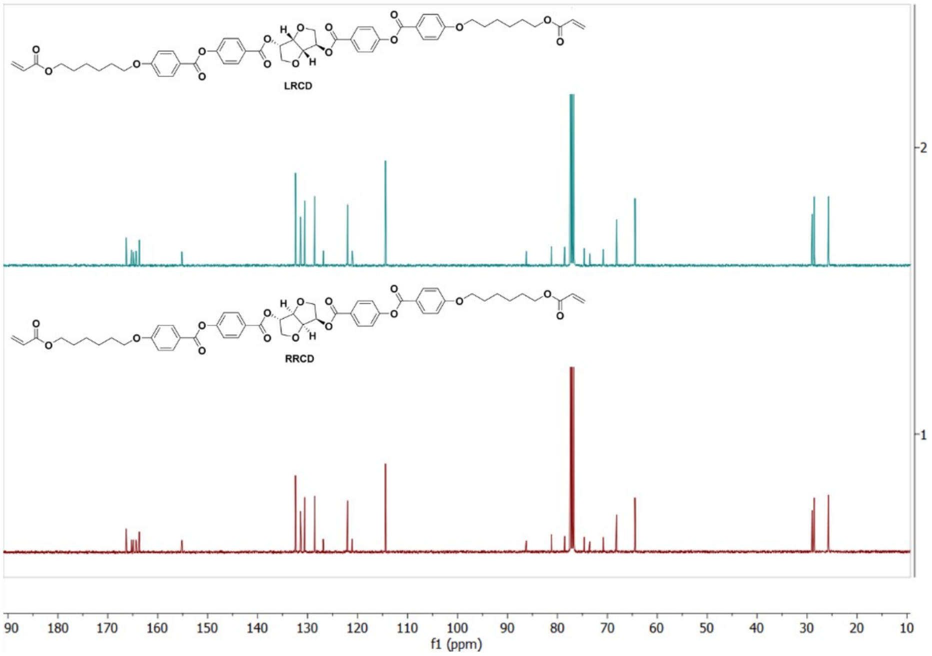

Figure S7: ^{13}C-NMR spectra of LRCD and RRCD in $CDCl_3$.

MALDI-TOF-MS spectra of LRCD and RRCD

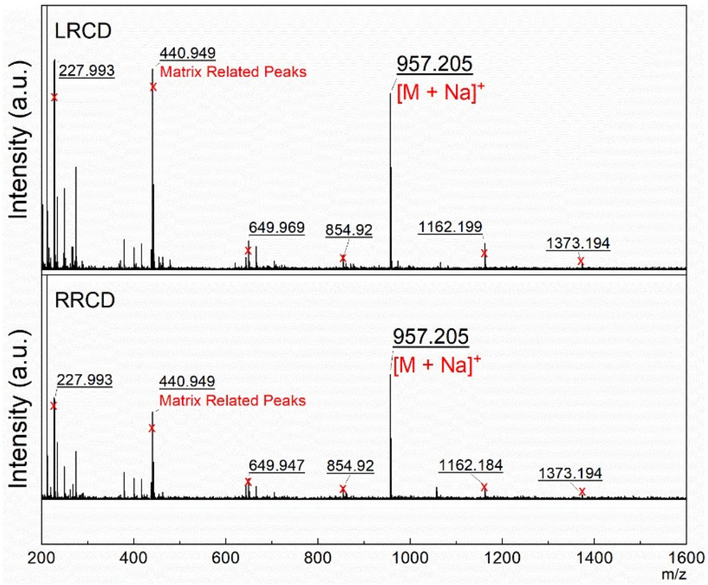

Figure S8: MALDI-TOF-MS full spectra of LRCD and RRCD with α-Cyano-4-hydroxycinnamic acid (CHCA) matrix. The matrix-related peaks are crossed in red.

Specific optical rotations of isosorbides and reactive chiral dopants

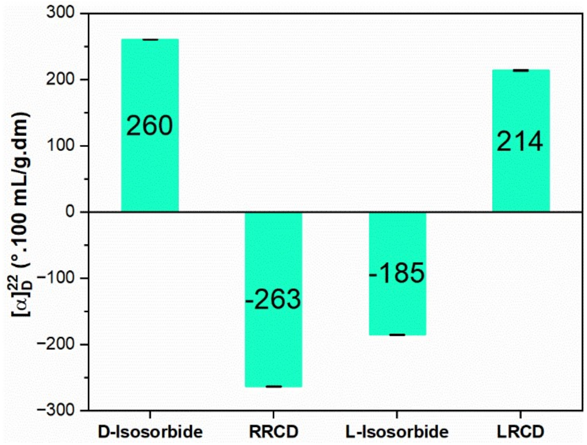

Figure S9 : Specific optical rotation measurements of D-Isosorbide (C=0.1, EtOH), RRCD (C=0.05, $CHCl_3$), L-Isosorbide (C=0.1, EtOH) and LRCD (C=0.05, $CHCl_3$), respectively.

LRCD doped thermochromic CLC coating UV-Vis-NIR transmission spectra

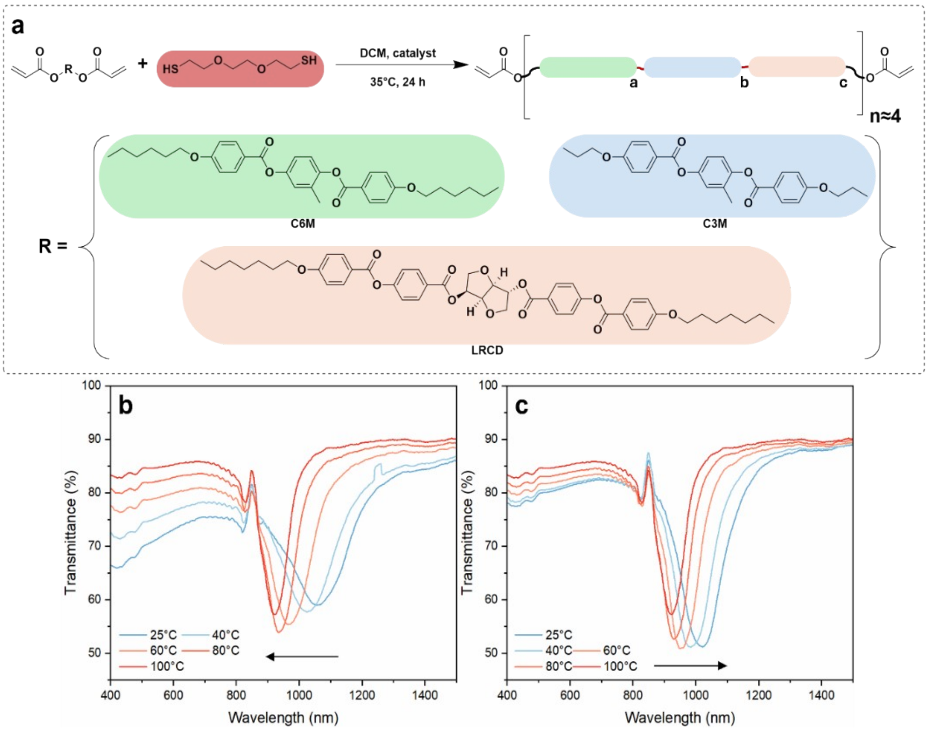

Figure S10 : a) Synthesis of a thermochromic CLC oligomer via a base catalyzed thiol-ene reaction[1]. b,c) UV-Vis-NIR spectra of LRCD doped thermochromic oligomer coating. b) Heating cycle. c) Cooling cycle.

1. Sentjens, H. *et al.* Programming Thermochromic Liquid Crystal Hetero-Oligomers for Near-Infrared Reflectors: Unequal Incorporation of Similar Reactive Mesogens in Thiol-ene Oligomers. *Macromolecules* **56**, 59–68 (2023).
